# Complete Genome Sequence of *Brucella abortus* SKN 13 Isolated from Placenta of Aborted Cattle in Gujarat, India

**DOI:** 10.1128/genomeA.01123-16

**Published:** 2016-10-27

**Authors:** H. C. Chauhan, Bharat K. Patel, B. S. Chandel, Kirit B. Patel, A. C. Patel, M. D. Shrimali, S. S. Patel, A. G. Bhagat, Manish Rajgor, Mitul A. Patel, Maulik Patel, Jitendra Kala, Bhumika Patel

**Affiliations:** Department of Animal Biotechnology, College of Veterinary Science and Animal Husbandry, Sardarkrushinagar Dantiwada Agricultural University, Sardarkrushinagar, Gujarat, India

## Abstract

*Brucella abortus* is generally known to cause brucellosis in cattle and buffalo. Here, we report the draft genome sequence of *Brucella abortus* SKN 13, isolated from aborted cattle placenta in the area of Gujarat, India, providing precious resources for comparative genomic analyses of *Brucella* field strains.

## GENOME ANNOUNCEMENT

Brucellosis is an important re-emerging zoonosis with worldwide distribution. It is still a serious uncontrolled public health problem in many developing countries including India (1). According to the Food and Agriculture Organization (FAO), the World Health Organization (WHO), and the World Organization of Animal Health (OIE), brucellosis is considered to be the most widespread zoonosis throughout the world (2). Brucellosis is a serious zoonotic disease that causes abortions, infertility, retention of placenta, and stillbirth in animals, leading to huge economic losses to the Indian dairy industry and farmers. Infectious diseases are a serious threat because of the rapidity with which they can spread and inflict losses in terms of mortality, morbidity, loss of production, etc. Brucellosis is an important reproductive disease of livestock and is considered to be a reemerging infectious disease in many areas of the world. Among the many names of brucellosis are Malta fever, undulant fever, gastric fever, and contagious abortion. 

*Brucella* bacteria are small (0.5 to 0.7 by 0.6 to 1.5 µm), nonmotile, noncapsulated coccobacilli. The different species of *Brucella* are genetically very similar, although each has slightly different host specificity. Different species of *Brucella* are *B. abortus*, *B. canis*, *B. ceti*, *B. melitensis, B. microti, B. neotomae, B. ovis, B. pinnipedialis*, and *B. suis* and these species have their preferred hosts, e.g., *B. abortus* infects cattle and *B. ovis* infect sheep, although they can also infect other animals (3).

The genomics DNA of *Brucella abortus* SKN 13 was isolated and sequenced using Ion Torrent Sequencing Technology with the provided standard protocol then assembled with MIRA version 3.9.18 by the *de novo* assembly method. The sequence is 3,277,130 bp with 52 contigs and a G+C content of 57.2% (4).

The genome sequence was then annotated with RAST and the NCBI Annotation Pipeline (http://www.ncbi.nlm.nih.gov/genome/annotation_prok/). A total of 3,192 genes were predicted, including 3,011 protein-coding genes, 51 tRNAs, three copies of 5SRNA, four copies of large subunit rRNA, and two copies of small-subunit rRNA (5). The draft genome sequences were compared with some closely related sequences predicted by RAST showing slight variations. These differences may result from adaptation or immune stress from the hosts.

### Accession number(s).

The draft genome sequence of *Brucella abortus* SKN 13 was submitted to NCBI under the accession numbers LIEA01000001 to LIEA01000052.
